# 

**Published:** 1904-06

**Authors:** 


SUBSCRIPTION $1 00

PUBLISHED MONTHLY

### ATLANTA JOURNAL-RECORD OF MEDICINE.

Successor to Atlanta Medical and Surgical Journal, Established 1855, and Southern Medical Record, Established 1870.

VOL. VI. ATLANTA, GA., JUNE, 1904. No. 3.

